# Supplementary material for: Immune Responses Induced by Recombinant Membrane Proteins of Mycoplasma agalactiae in Goats
Source: Vaccines (Basel). 2025 Jul 11;13(7):746. doi: 10.3390/vaccines13070746 (PMC12299757; doi:10.3390/vaccines13070746)
Supplement: Supplementary file 1 [file vaccines-13-00746-s001.zip › vaccines-3687753-supplementary.pdf]

vaccines

MDPI

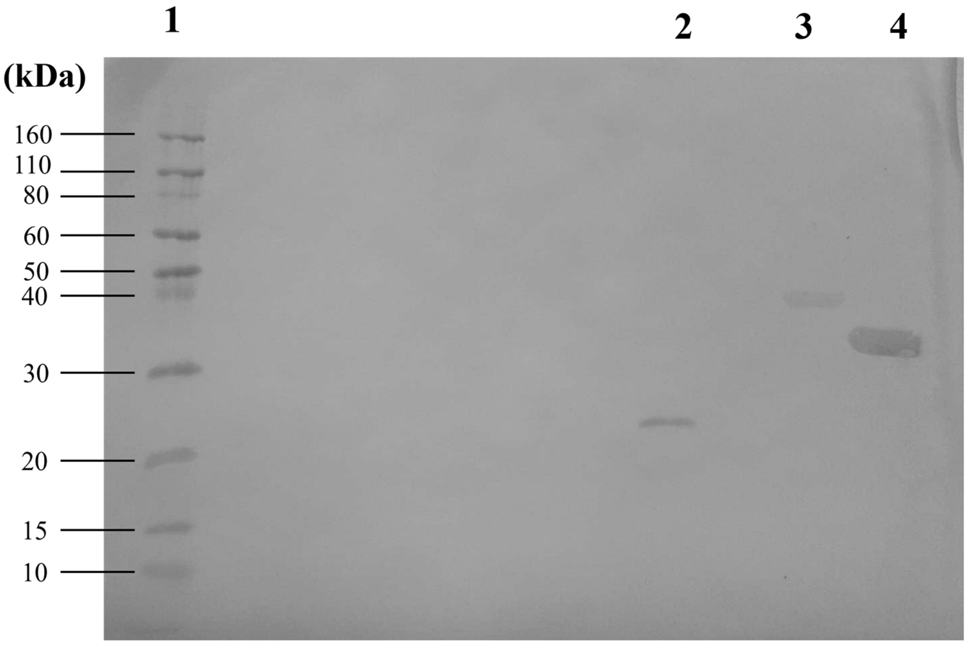

Figure S1. Recombinant proteins visualized by Western blotting. Column 1: Ladder: Novex® Sharp Pre-stained Protein Standard; Column 2: unrelated protein from our lab (58,91µg; densitometry ratio: 1); Column 3: P40 (42 kDa – densitometry ratio: 1,24); Column 4: MAG_1560 (32 kDa – densitometry ratio: 5,28).

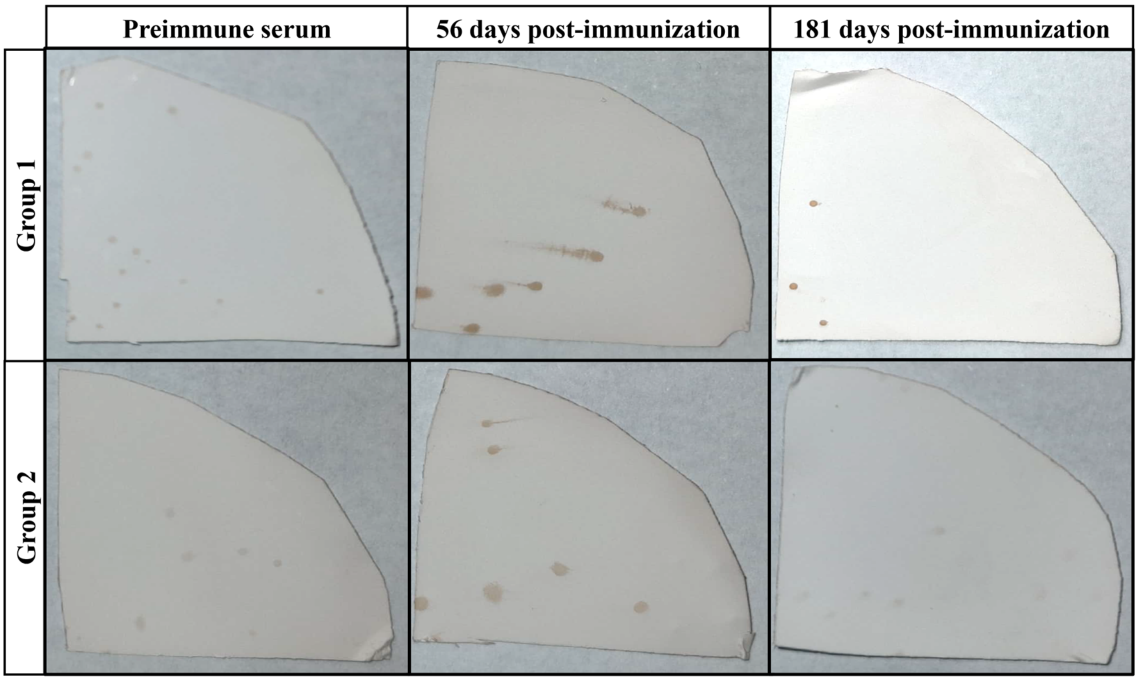

Figure S2. Complete detection membranes of *M. agalactiae* colonies by colony immunoblotting. Detection of *M. agalactiae* strain GM139 colonies by specific antibodies present in the serum of group 1 and group 2 animals at pre-immunization and 56 and 181 days post-immunization. Recognition is indicated by increased staining intensity, represented by the brown coloration.
